# Towards Laser-Textured Antibacterial Surfaces

**DOI:** 10.1038/s41598-018-28454-2

**Published:** 2018-07-04

**Authors:** Adrian H. A. Lutey, Laura Gemini, Luca Romoli, Gianmarco Lazzini, Francesco Fuso, Marc Faucon, Rainer Kling

**Affiliations:** 1 Università degli studi di Parma, Dipartimento di Ingegneria e Architettura, Parma, 43124 Italy; 2 ALPhANOV Centre Technologique Optique et Lasers, Talence, 33400 France; 3 Università di Pisa, Dipartimento di Fisica Enrico Fermi, Pisa, 56127 Italy

## Abstract

*Escherichia coli* and *Staphylococcus aureus* bacterial retention on mirror-polished and ultrashort pulse laser-textured surfaces is quantified with a new approach based on ISO standards for measurement of antibacterial performance. It is shown that both wettability and surface morphology influence antibacterial behavior, with neither superhydrophobicity nor low surface roughness alone sufficient for reducing initial retention of either tested cell type. Surface structures comprising *spikes*, laser-induced periodic surface structures (*LIPSS*) and *nano-pillars* are produced with 1030 nm wavelength 350 fs laser pulses of energy 19.1 μJ, 1.01 μJ and 1.46 μJ, respectively. SEM analysis, optical profilometry, shear force microscopy and wettability analysis reveal surface structures with peak separations of 20–40 μm, 0.5–0.9 μm and 0.8–1.3 μm, average areal surface roughness of 8.6 μm, 90 nm and 60 nm and static water contact angles of 160°, 119° and 140°, respectively. *E*. *coli* retention is highest for mirror-polished specimens and *spikes* whose characteristic dimensions are much larger than the cell size. *S*. *aureus* retention is instead found to be inhibited under the same conditions due to low surface roughness for mirror-polished samples (*S*_*a*_: 30 nm) and low wettability for *spikes*. *LIPSS* and *nano-pillars* are found to reduce *E*. *coli* retention by 99.8% and 99.2%, respectively, and *S*. *aureus* retention by 84.7% and 79.9% in terms of viable colony forming units after two hours of immersion in bacterial broth due to both low wettability and fine surface features that limit the number of available attachment points. The ability to tailor both wettability and surface morphology via ultrashort pulsed laser processing confirms this approach as an important tool for producing the next generation of antibacterial surfaces.

## Introduction

The ability to create passive antibacterial surfaces on industrial components has profound implications for human health services and food production^1–3^. To achieve this, an appropriate scalable technology must be identified based on clear parameters for reducing biofouling under real operating conditions. The self-cleaning lotus leaf has inspired attempts to mimic hierarchical micro and nanoscale surface structures responsible for superhydrophobicity and removal of contaminants via the rolling action of individual water droplets, the so-called “lotus effect”^4–8^. Other approaches have examined surface structures found on insects that promote self-cleaning and direct rupture of bacterial cell membranes^9–11^. Ultrashort pulsed laser irradiation is a strong candidate for producing surfaces of this kind due to the reliability with which nano- and micro-scale features can be created over large areas with a single process^12–16^. Superhydrophobic and antibacterial laser-textured surfaces have been demonstrated in the literature^17,18^; however, the relative importance of wettability and morphology in reducing bacterial retention in this context has not always been distinguished. Differentiation of these aspects for laser-textured surfaces is important from a process point of view as the feasibility of upscaling for high throughput is strongly influenced by the choice of process parameters and therefore optimization strategy. The effects of wettability and morphology on bacterial retention in the context of laser-textured surfaces must therefore be more clearly established to provide precise guidelines for future manufacturing of antibacterial surfaces.

The ability of a bacterial cell to remain attached to a surface reflects the nature of non-covalent interactions between the substrate and cell wall functional groups during the initial phases of attachment. According to attachment point theory, organisms smaller than the characteristic surface feature size can achieve higher attachment strength due to the large available contact area and sheltering effect of surface roughness, while those that are not are more limited in their ability to remain in contact with the surface^19^. Such a representation is based on direct interaction between the substrate and cell wall, where cell stiffness limits its capacity to adapt to very small surface features^20,21^. Experimental investigations have shown that retention is strongly limited where the spacing between surface features is smaller than the cell size^19,22^. This effect has been shown to be particularly evident for engineered surfaces with well-defined features and dimensions^23^. In some cases, fine surface features are more effective at reducing bacterial retention than extremely smooth surfaces, where biofilm formation has been shown to increase for very low roughness^24^. The preferential alignment of microorganisms to valley and pillar structures demonstrates that cells seek to maximize contact area for improved attachment^25–27^. It is therefore expected that the presence of small features relative to cell size can reduce cell retention by limiting the number of attachment points.

The role of wettability on cell attachment, adhesion and retention has been demonstrated for polymers and impregnated fabrics whose static water contact angle is modified via changes in chemical composition^28–30^. It has been shown that superhydrophobic surfaces produced by aerosol-assisted chemical vapor deposition can reduce *Escherichia coli* (*E*. *coli*) and *Staphylococcus aureus* (*S*. *aureus*) cell attachment by up to 79% and 63%, respectively^31^. Superhydrophobic and superhydrophilic surfaces with identical surface morphology have been shown to create regions of preferential attachment for specific cell types^32^. *Micromonospora purpurea* adhesion has also been found to decrease for duplicated dried-gel superhydrophobic surfaces with a static water contact angle of 150° compared to surfaces with the same topography and contact angles in the range 54°–130°^33^.

In studies relating to laser-treated metallic surfaces, wettability has been shown to be of lesser influence on bacterial retention than surface morphology for certain cell types. Laser-textured titanium surfaces with micro-bumps larger than 10 µm have been shown to favor *S*. *aureus* cell attachment despite a static water contact angle of 166°^18^. Colonization of *Pseudomonas aeruginosa* has instead been found to diminish under the same conditions. Laser-textured stainless steel surfaces with laser-induced periodic surface structures (*LIPSS*) of period 0.7 µm yielded contrasting results for *E*. *coli* and *S*. *aureus*, with bacterial retention decreasing in the former case and increasing in the latter compared to untreated samples with the same wettability^17^. Reductions in *S*. *aureus* have instead been observed for hydrophilic titanium surfaces with *LIPSS* and *nano-pillars*^34^. Recent studies have found that surface roughness, morphology, chemistry and wettability all play a part in *E*. *coli* attachment, adhesion and retention on *LIPSS* produced via picosecond pulsed laser irradiation^35,36^. These works have also shown that surfaces displaying lowest bacterial adhesion are not necessarily the most hydrophobic. Dependence of bacteria retention on surface structure dimensions has furthermore been demonstrated for fine surface structures produced via direct laser interference patterning (DLIP), irrespective of surface wettability^37,38^. Though it is clear that wettability plays a role in influencing cell attachment where other properties remain unchanged, adhesion, retention and biofilm formation on laser-treated metallic surfaces with well-defined surface morphology appears to be strongly dependent on cell type and surface structure size.

Ultrashort pulsed laser irradiation is a well-established method for producing changes in surface morphology and wettability on metallic and semi-conductor surfaces. Interference effects between laser radiation and resulting surface plasmon polaritons on time-scales shorter than the electron-phonon relaxation time lead to formation of self-organized *LIPSS* with surface feature dimensions close to the laser wavelength for low spatial frequency *LIPSS* and down to one tenth of the wavelength or smaller for high spatial frequency *LIPSS*^12,39^. The physical mechanisms responsible for *LIPSS* have received extensive attention in the literature^13^. With increasing energy dose, the effects of ablation, heat accumulation and hydrodynamic instability lead to hierarchical micro-scale *bumps* and *spikes* with superimposed *LIPSS*^12,15,40–42^. It has been proposed that ultrashort pulsed laser irradiation of stainless steel also leads to oxygen deficiency in the form of active magnetite, which is initially hydrophilic before becoming hydrophobic over a period of several weeks due to dissociative absorption of carbon dioxide from air^43^. It has been observed experimentally that this transition can be inhibited by submerging the treated surface in boiling water for 48 hours^43^. Laser parameters and post-treatment procedures can therefore be tailored to achieve a wide range of surface structures and wettability characteristics that may be appropriate for creating antibacterial surfaces in healthcare and food production machinery.

The present work clarifies factors influencing *E*. *coli* and *S*. *aureus* retention on laser-textured specimens by quantifying the performance of several representative surface structures and examining the individual effects of wettability, morphology and topography through discrete manipulation and analysis of these properties. Mirror-polished and superhydrophobic laser-textured surfaces with large surface features are firstly produced to demonstrate limitations associated with low surface roughness and low wettability alone. Laser-textured surfaces with fine features of similar size to bacterial cells are then produced with the aim of minimizing available attachment points. Hydrophobic and hydrophilic specimens with the same surface texture are both considered by ageing laser-treated samples in different environments. Bacterial retention is then assessed based on ISO standards for measurement of antibacterial performance and sampling techniques using contact plates and swabs. Tests are performed with upward-facing horizontal specimens and fluid agitation to ensure bacterial cells are continuously brought into contact with the surface during the exposure period and are not limited to the fluid-surface interface^44^. Wettability, SEM and topography analyses are performed on all specimens to correlate initial bacterial retention with distinct surface properties. The advantages of this approach are two-fold; firstly, the effects of wettability, morphology and topography on bacterial retention are differentiated for laser-textured stainless steel surfaces, allowing emphasis to be placed on development of laser technology and scanning strategies that favor characteristics directly influencing bacterial retention. Secondly, a measurement approach for antibacterial performance is developed based on ISO standards already of widespread use within the food production industry to facilitate direct technology transfer to end-users. Further to demonstrating that both wettability and morphology are fundamental in driving biofilm formation over a wide spectrum of cell types, the present work confirms laser technology as a valid approach for manufacturing the next generation of antibacterial surfaces.

## Materials and Methods

### Sample preparation

316 L stainless steel samples supplied by LIMDES were employed for all experiments. Specimens were 50 mm (w) × 50 (l) mm × 2 (h) mm in size and mechanically polished by the supplier to an average areal surface roughness (*S*_*a*_) of 30 nm. Additional polished samples were also utilized directly for bacterial retention tests to assess the effects of low surface roughness on biofilm formation. Control specimens were rolled 316 L stainless steel with an average areal surface roughness of 0.37 µm, chosen to represent present-day practices within the food handling industry and meet current industry standards^45^.

### Laser treatment

Laser irradiation was performed with an Amplitude Systemes Satsuma HP3 laser source with emission wavelength of 1030 nm and pulse duration of 350 fs. The laser output was firstly directed through a polarizer and half-wave plate for fine adjustment of the laser pulse energy, prior to a beam expander to achieve an appropriate beam size. A galvanometric scanning head and a 100 mm focal length f-theta lens were then employed to focus the laser beam on the specimen surface. *LIPSS*, *spikes* and *nano-pillars* were generated over the entire 50 mm × 50 mm surface of each individual specimen utilizing the process parameters given in Table 1, which had been optimized in a previous work^40^. Specimens were aged in ambient air for 30 days to ensure complete transition from a hydrophilic to a hydrophobic state^40,43^. An additional set of *LIPSS* samples was prepared and held in water at 90 °C for 48 hours immediately following laser treatment to achieve a permanent hydrophilic state^43^.

**Table 1:** Laser parameters utilized in experiments.

Parameter	*Spikes*	*LIPSS*	*Nano-pillars*
Polarization	Linear	Linear	Azimuthal
Repetition rate (kHz)	1000	1000	250
Pulse energy (µJ)	19.1	1.01	1.46
Scanning velocity (m/s)	2	1	1
Hatch spacing (µm)	5	5	3
Number of passes	10	1	1
Energy dose (J/cm^2^)	1910	20.2	12.2

### Morphology and topography

All laser-treated samples were analyzed with a scanning electron microscope (SEM) to confirm their morphology and assess the resulting surface structures. Topography measurements were carried out on *spikes* with a Taylor Hobson Talysurf CCI optical profiler equipped with a 50× objective. This configuration gave a 336 µm × 336 µm sampling area with nominal horizontal and vertical resolutions of 0.4 µm and <1 nm, respectively. Topography measurements were instead carried out on *LIPSS* and *nano-pillars* with shear force microscopy (ShFM), a high-resolution non-contact scanning probe technique that was necessary due to the fine surface features of these specimens. The ShFM setup comprised an electrochemically etched tungsten wire with a nominal diameter of 50 nm joined to a quartz tuning fork with adhesive and maintained in oscillation parallel to the sample surface at 32 kHz. A RHK SPM-100 controller and Physik Instrumente P-517.3 three-axis nano-positioner with horizontal and vertical resolutions of 1 nm and 0.1 nm, respectively, were utilized to scan a sampling area of up to 100 µm × 100 µm while maintaining the tip within a few nanometers of the surface. The oscillation amplitude and phase resulting from friction within the air layer between the tip and surface during ShFM were monitored continuously and applied in a feedback loop to measure the profile in a non-contact manner^46,47^.

Areal topography parameters, including average areal surface roughness (*S*_*a*_), skewness (*S*_*sk*_), kurtosis (*S*_*ku*_) and density of peaks (*S*_*pd*_), were calculated in accordance with ISO 25178^48^ based on the acquired topography data from the optical profiler and ShFM analysis. *S*_*a*_ represents a commonly used parameter to assess the average deviation of the local surface height from the mean plane^21^. It is known, however, that the average roughness is not sufficient to uniquely describe a surface in relation to bacterial attachment^49,50^. *S*_*sk*_ and *S*_*ku*_, representing the second and third order moments of the height distribution over the evaluation area, were therefore also determined. *S*_*pd*_ is instead defined as the number of peaks per unit surface area. For calculation of this parameter, application of the watershed algorithm was required to segment the surface into hills or motifs. All relevant definitions for calculation of topography parameters are given in ISO 25178^48^.

### Wettability

Surface wettability was quantified in terms of the static water contact angle and sliding angle^51^ as measured via the sessile drop method with a Dataphysics Instruments OCA20 goniometer. A water droplet volume of 6 µL and syringe diameter of 310 µm were employed for all tests. Sliding angle measurements were performed with a stationary deposited droplet at a rotation rate of 1 °/s. The sliding angle was taken as the angle at which the droplet completely detached from the surface.

### Bacterial retention

*E*. *coli* and *S*. *aureus* were chosen as representative rod-like gram-negative and spherical gram-positive bacteria strains, respectively. Cell retention tests were performed on three identical 50 × 50 mm samples for all combinations of surface type and bacteria. Additional tests were performed with *E*. *coli* on hydrophilic *LIPSS* that had been held in hot water for 48 hours immediately after laser treatment. Finally, hydrophobic *LIPSS* subject to prior contamination and sterilization were tested a second time to assess the robustness of the developed treatments. An equal number of control samples of the same size were tested simultaneously in the same bacteria solutions to provide reference values, monitor solution stability and account for possible differences in bacteria density. Control samples had a surface roughness of 0.37 µm, chosen to represent present-day practices within the food handling industry and meet current industry standards for food handling equipment^45^.

The method employed for quantifying bacterial retention was developed based on ISO 22196^52^ and ISO 27447^53^ standards for measurement of antibacterial performance. Samples were ultrasonically cleaned in acetone for 15 minutes before being sterilized in pure ethanol for 10 minutes and dried in a UV hood until all residue was eliminated. *E*. *coli* (ATCC 8739) and *S*. *aureus* (ATCC 6538 P) colonies supplied by Microbiologics were transferred separately into Nutrient Agar (NA) with inoculation loops and incubated for 24 hours at 37 °C before being transferred into NA and incubated for a further 18 hours. This procedure was performed in line with ISO 27447 to ensure that the bacteria were in an appropriate growth phase and free from environmental stress. The stock was then diluted to N/500 with an optical density of 0.5 to achieve isotonic cell properties and allow transfer from NA to solution. The resulting fluid was transferred into 200 mL sterile containers in which individual samples were submerged and held horizontally with the laser-treated surface facing upwards for two hours at 24 °C. During this period, containers were agitated with a translating mechanical agitator at a frequency of 1.5 Hz and a stroke of 30 mm to ensure bacterial cells were continuously brought into contact with the surface^44^. The cell density of fluids employed for each set of tests is given in Table 2. Upon completion, samples were held in a vertical position for 120 s to remove excess liquid from the surface. Residual micro-organism numbers were then quantified in line with ISO 18593 for horizontal sampling methods. Orthogonal surface swabs were taken from each sample over an area of 50 × 40 mm, diluted in solution and seeded in NA prior to incubation at 37 °C for 48 hours. Colony forming units per swab (cfu/swab) were then quantified with a colony counter for each sample.

**Table 2:** Cell density of fluids employed for each set of tests.

Parameter	*Mirror-Polished*	*Spikes*	*H.phob LIPSS* ^*^*^	*H.phil LIPSS* ^*^*^	*Nano-pillars*
Cell density, *E. coli* (cfu/ml)	2.4 × 10^7^	1.3 × 10^7^	1.3 × 10^7^	2.4 × 10^7^	2.6 × 10^7^
			2.4 × 10^7^ (Repeat^*^)		
Cell density, *S. aureus* (cfu/ml)	3 × 10^6^	7 × 10^6^	7 × 10^6^	—	7 × 10^6^

^^^*H*.*phob LIPSS* refers to samples aged in ambient air for 30 days; *H*.*phil LIPSS* refers to samples held in water at 90 °C for 48 hours. ^*^H.*phob LIPSS repeat* refers to samples aged in ambient air for 30 days and contaminated a second time with *E*. *coli*.

### Data availability

The datasets generated during and/or analyzed during the current study are available from the corresponding author on reasonable request.

## Results

### Laser treatment

**Figure 1:**
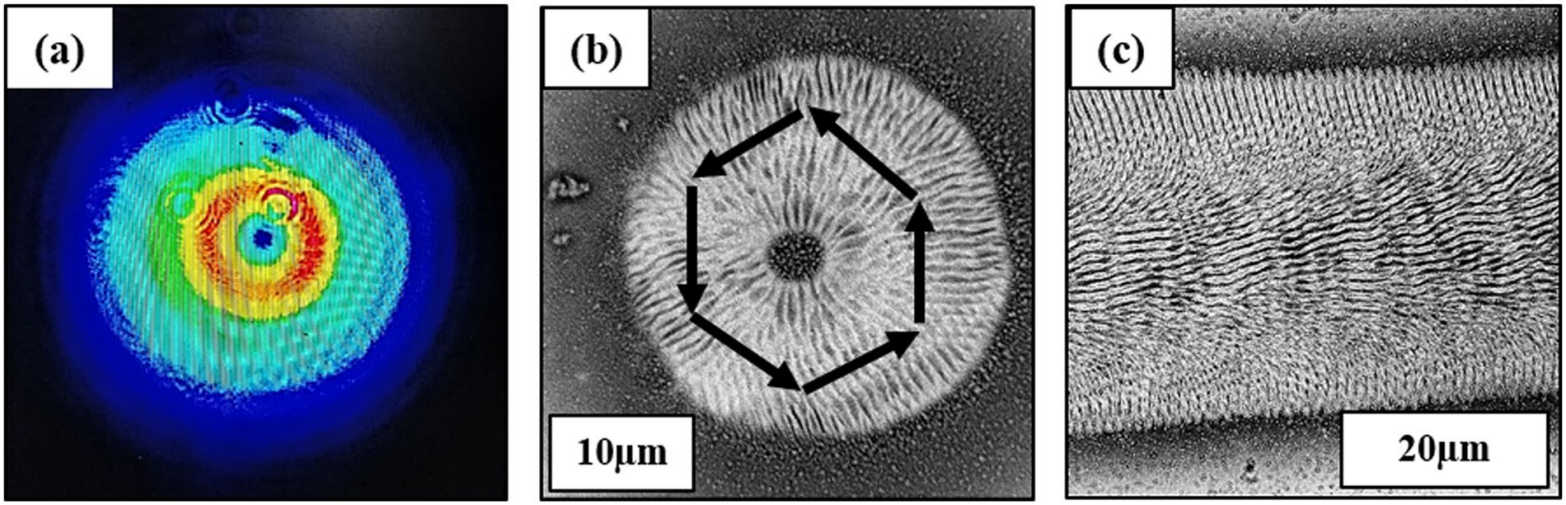
(**a**) Beam shape after s-waveplate polarizer as recorded by CDD camera (**b**) surface structure produced on steel after irradiation with azimuthal polarized beam and (**c**) scanned line with azimuthal polarized beam at 250 kHz repetition rate.

Laser exposure with the parameters given in Table 1 successfully led to development of *spikes*, *LIPSS* and *nano-pillars*. As reported in the literature, *LIPSS* and *spikes* are easily obtained via ultrashort pulsed laser irradiation. The characteristics of *LIPSS* are strongly dependent on the properties of the incident laser beam, including the polarization orientation and wavelength. Linearly polarized ultrashort laser pulses produce *LIPSS* that are perpendicular to the polarization orientation. *Spikes* develop at higher energy dose due to the onset of ablation, heat accumulation effects and hydrodynamic instability^12,15,40–42^. *Nano-pillars* instead exploit the possibility of modifying *LIPSS* geometry by varying the polarization type^14^. Figure 1a presents a CDD camera image of the beam shape obtained with azimuthal polarization (Altechna s-waveplate polarizer). The initial Gaussian beam was converted into a donut shape that produced the surface structure shown in Fig. 1b. *LIPSS* extended from the center of the beam to the border, perpendicular to the laser light polarization direction (indicated for several positions with arrows). Figure 1c shows a representative line obtained on samples after scanning with this setup at 1 m/s and a repetition rate of 250 kHz. It is possible to observe that the resulting *LIPSS* have different directions at the edges of the line than at the center. When irradiating several subsequent lines with a specific separation hatch distance (Table 1), the geometric intersection between each set of *LIPSS* led to the formation of distinct *nano-pillars* for a given set of process parameters.

### Morphology and topography

**Figure 2:**
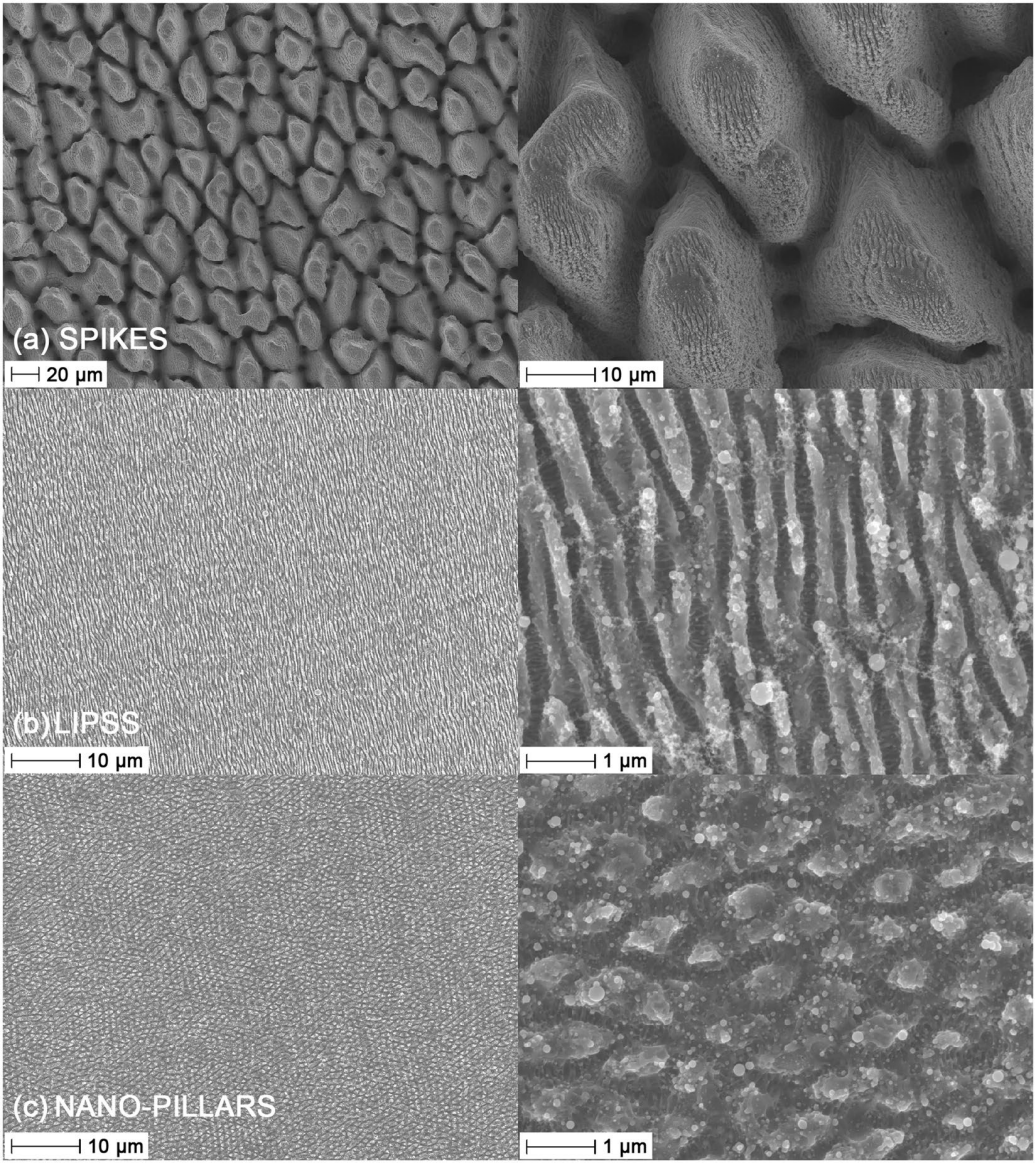
SEM images of laser-treated surfaces: (**a**) *Spikes* (left: 1000×, right: 5000×), (**b**) *LIPSS* (left: 5000×, right: 50000×), (**c**) *Nano-pillars* (left: 5000×, right: 50000×).

**Figure 3:**
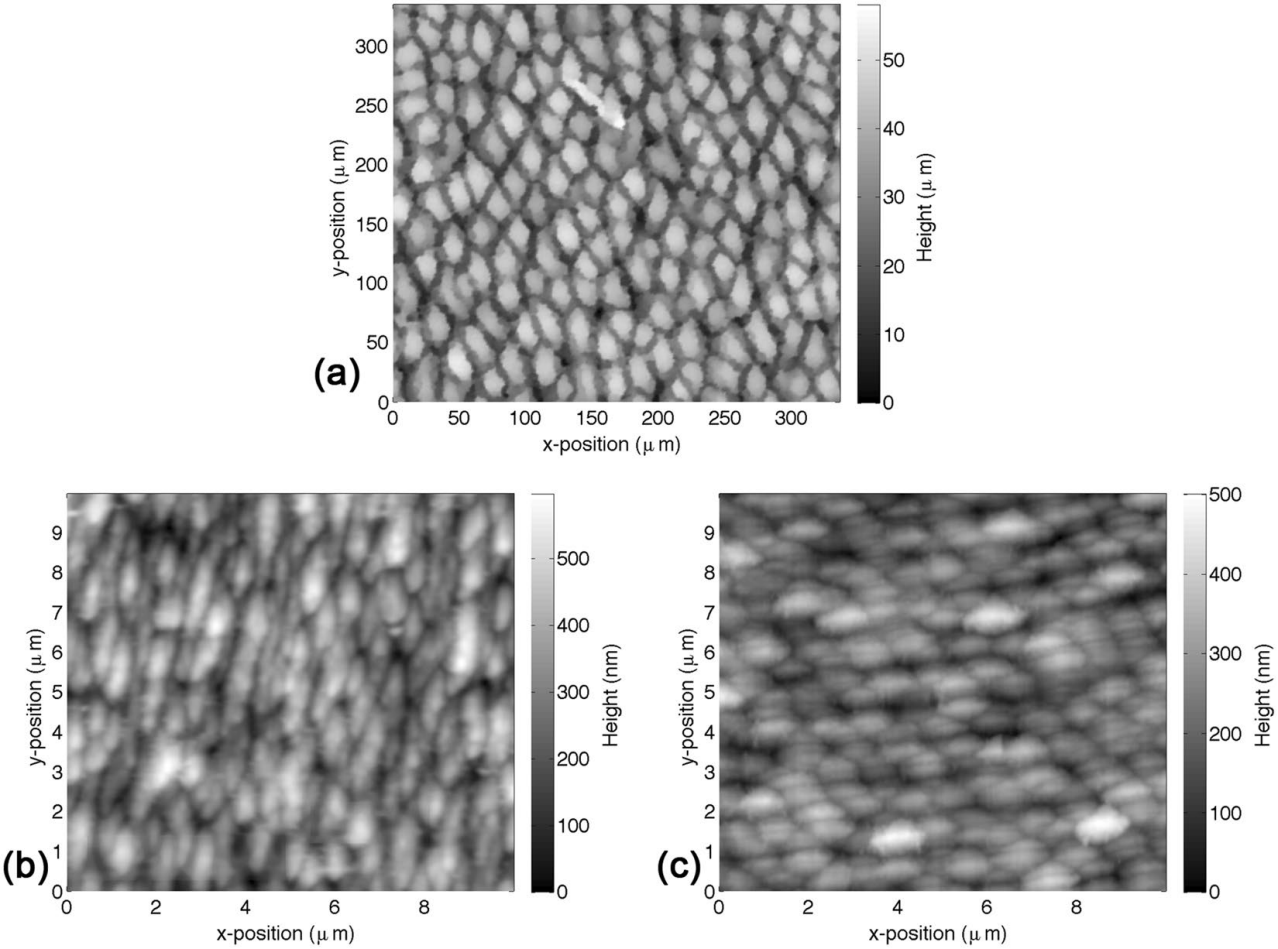
Surface topography of laser-treated surfaces: (**a**) *Spikes* (acquired with optical profiler), (**b**) *LIPSS* (acquired with ShFM) and (**c**) *Nano-pillars* (acquired with ShFM).

SEM images and topography maps of the laser-treated surfaces are presented in Figs 2 and 3. Areal surface roughness parameters are given in Table 3 for all tested surfaces, including control and mirror-polished specimens. Large differences in dimensions can be observed between each laser-textured structure type in terms of lateral dimensions, topography and form. The attainment of *LIPSS* following ultrashort pulsed laser irradiation with linear polarization at low energy dose and *spikes* at high energy dose is in line with the literature^12,15,40–42^. These characteristics were sought after to directly assess the effects of surface morphology on bacterial retention. *Spikes* produced at high energy dose were characterized by elongated asperities or homogeneous columnar structures with a peak separation of 20–40 µm and an average areal surface roughness of 8.6 µm. Skewness (−0.10) and kurtosis (2.0) indicated an even height distribution. *LIPSS* produced at low energy dose were characterized by elongated parallel ridges perpendicular to the laser polarization orientation with a ridge separation of 0.5–0.9 µm. *Nano-pillars* presented a relatively homogeneous surface with a peak separation of 0.8–1.3 µm. Average areal surface roughness was 90 nm for *LIPSS* and 60 nm for *nano-pillars*, marginally higher than mirror-polished specimens (30 nm). Skewness (*LIPSS*: 0.13, *nano-pillars*: 0.54) and kurtosis (*LIPSS*: 2.9, *nano-pillars*: 3.9) indicated a relatively symmetrical height distribution for *LIPSS* and a prevalence of sharp peaks for *nano-pillars*. Average areal surface roughness for control samples was 0.37 µm.

**Table 3:** Measured topography parameters for control, mirror-polished and laser-treated surfaces.

Parameter	*Control*	*Mirror-Polished*	*Spikes*	*LIPSS*	*Nano-pillars*
Areal roughness, S_a_ (nm)	370 ± 40	30 ± 5	8600 ± 100	90 ± 5	60 ± 5
Skewness^^^, S_sk_	—	—	−0.10 ± 0.01	0.13 ± 0.05	0.54 ± 0.05
Kurtosis^^^, S_ku_	—	—	2.0 ± 0.1	2.9 ± 0.2	3.9 ± 0.2
Density of peaks^^^, S_pd_ (µm^−2^)	—	—	0.0017 ± 0.0002	1.1 ± 0.1	1.3 ± 0.1

^^^Skewness, kurtosis and density of peaks calculated for laser-treated samples only.

### Wettability

After 30 days of ageing in ambient air, *spikes* were superhydrophobic with a static water contact angle of 160° and a sliding angle of 14°. This outcome is consistent with other works, where a transition from a hydrophilic to a superhydrophobic state has been observed over several weeks following ultrashort pulsed laser irradiation of stainless steel^43^. The wettability of aged *LIPSS* and *nano-pillars* was somewhat higher than *spikes*, with static contact angles of 119° and 140°, respectively. Water droplet sliding did not take place on these specimens for angles up to 90°. *LIPSS* and *nano-pillars* were therefore hydrophobic but not superhydrophobic. The static contact angle of hydrophilic *LIPSS*, held in water at 90 °C for 48 hours following laser irradiation, was instead 26°. This result is consistent with the findings of Kietzig *et al*.^43^, who suggested that oxygen ions from water deactivate active magnetite sites to form hydrophilic iron oxides. All measured wettability data are provided in Table 4.

**Table 4:** Measured static water contact angle and sliding angle for laser-treated surfaces.

Parameter	Spikes	LIPSS	Nano-pillars
Static water contact angle (°)	160 ± 6	119 ± 5 (H.phob^^^) 26 ± 5 (H.phil^)	140 ± 2
Water sliding angle (°)	14 ± 3	—	—

^^^*H.phob LIPSS* refer to samples aged in ambient air for 30 days; *H.phil LIPSS* refer to samples held in water at 90 °C for 48 hours following laser treatment.

### Bacterial retention

**Figure 4:**
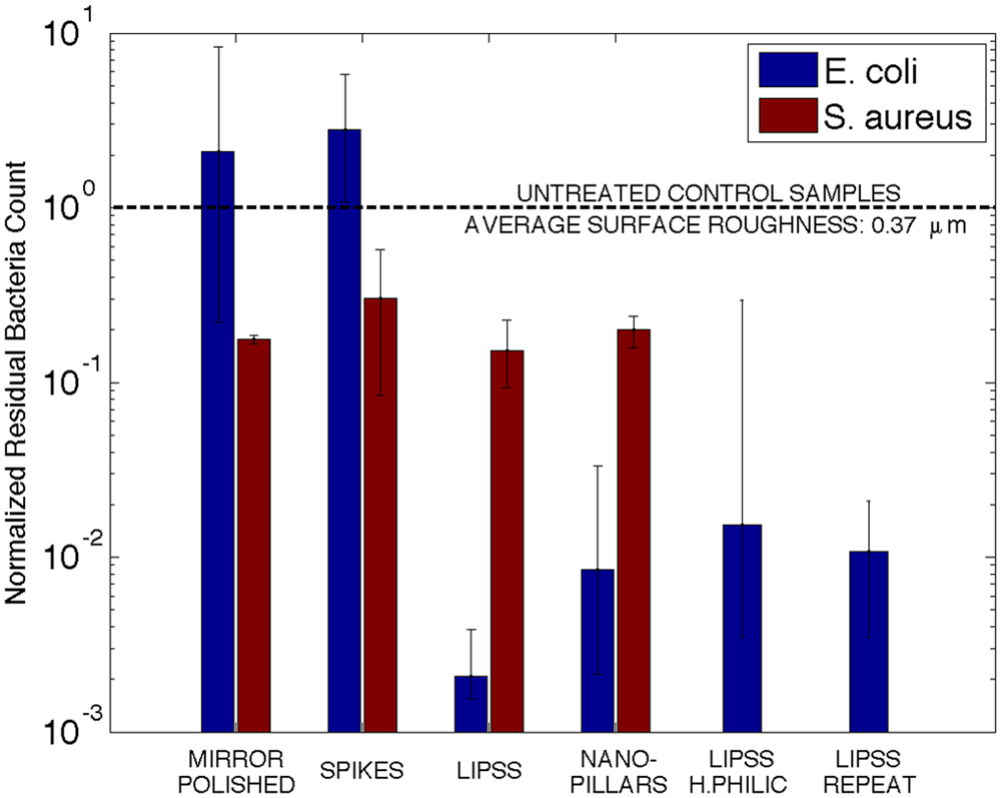
Geometric average of normalized residual *E*. *coli* and *S*. *aureus* bacteria counts for mirror-polished and laser-treated surfaces with *spikes*, *LIPSS* and *nano-pillars*. Three variations of *LIPSS* are presented: hydrophobic specimens contaminated separately with *E*. *coli* and *S*. *aureus* (*“LIPSS”*), hydrophilic specimens contaminated with *E*. *coli* (“*LIPSS H*.*PHILIC*”) and hydrophobic samples contaminated a second time with *E*. *coli* (“*LIPSS REPEAT*”). Values are normalized against residual bacteria counts for untreated control samples (*S*_*a*_: 0.37 µm) tested under the same conditions, chosen to represent present-day practices within the food handling industry and meet current industry standards for stainless steel surfaces^45^. Error bars display maximum and minimum measured values.

The geometric average of normalized residual *E*. *coli* and *S*. *aureus* bacteria counts are shown in Fig. 4 for all tested surfaces. The developed method based on ISO 22196 and ISO 27447 was effective at quantifying bacterial retention over a diverse range of conditions. Results are normalized against control samples tested under the same conditions; values below unity represent a reduction in bacterial retention compared to present-day practices and values above unity represent a worse outcome. Results are presented on a logarithmic scale to better account for large differences in performance between surface types. Mirror-polished surfaces display strong dependence on bacteria type. These specimens performed worse than untreated samples for *E*. *coli*, with cell numbers almost doubling compared to the control samples. The same surface structure instead yielded an 82.4% reduction in *S*. *aureus*. Similarly, laser-induced *spikes* strongly promoted *E*. *coli* retention, with an almost three-fold increase in residual bacteria count. The same structure was instead found to reduce *S*. *aureus* retention by 69.8%. *LIPSS* and *nano-pillars* exhibited low residual bacteria counts for both tested cell types, with very large reductions attained for *E*. *coli*. Best performance was achieved with *LIPSS*, yielding reductions of 99.8% for *E*. *coli* and 84.7% for *S*. *aureus*. *Nano-pillars* performed similarly, with reductions of 99.2% and 79.9%, respectively. Hydrophilic *LIPSS* instead exhibited a 98.5% reduction in *E*. *coli* cell numbers but somewhat greater data dispersion. Finally, hydrophobic *LIPSS* samples subject to a second contamination cycle exhibited a 99.1% reduction in *E*. *coli* retention compared to the control samples, indicating a minor reduction in antibacterial performance compared to initial exposure (99.8%) but nonetheless robust behavior.

## Discussion

### Morphology and topography

The laser-treated surfaces can be divided into two broad categories based on their morphology (Fig. 2); *spikes* that present features much larger than bacterial cells (20–40 µm), and *LIPSS* and *nano-pillars* that present features similar in size (0.5–0.9 µm and 0.8–1.3 µm, respectively). The density of peaks, *S*_*pd*_, quantified during topography analysis (Table 3), can be considered a simple indicator of the number of available contact points for bacterial cells based on their dimensions. Rod-shaped *E*. *coli*, with a diameter of approximately 0.5 µm and a length of approximately 2 µm, occupy approximately 1 µm^2^/cell. A reduction in the number of contact points is therefore expected for *S*_*pd*_ > 1 µm^−2^. It has been shown that larger structures (*S*_*pd*_ < 1 µm^−2^) lead to a reduction in *E*. *coli* coverage^54^, for which the criterion *S*_*pd*_ > 1 µm^−2^ is a conservative indicator for reducing the number of available attachment points. Spherical *S*. *aureus* cells, with a diameter of approximately 0.5 µm, instead occupy approximately 0.25 µm^2^/cell. A reduction in the number of contact points in this case is therefore expected for *S*_*pd*_ > 4 µm^−2^. Though such values are based purely on geometric considerations and do not account for asymmetry of the cell or surface, they provide approximate threshold values that can be compared with readily measurable topography data. In the case of *spikes*, the density of peaks is 1.7 × 10^−3^ µm^−2^ (Table 3), some three orders of magnitude lower than the threshold value for *E*. *coli*. The density of peaks of *LIPSS* and *nano-pillars* are instead 1.1 µm^−2^ and 1.3 µm^−2^, respectively (Table 3), greater than the threshold value of 1 µm^−2^ for *E*. *coli* but below 4 µm^−2^ for *S*. *aureus*.

### Wettability

The laser-treated surfaces can also be divided in terms of wettability based on the measured static water contact angle and water sliding angle (Table 4); *spikes* that are superhydrophobic (contact angle of 160° and sliding angle of 14°), *LIPSS* and *nano-pillars* that are hydrophobic (contact angles of 118° and 140°, respectively, with no droplet sliding), and *LIPSS* held in hot water that are hydrophilic (contact angle of 26° with no droplet sliding). *Spikes* are therefore superhydrophobic but with a large feature size, a configuration chosen to allow assessment of the prevalence of morphology or wettability on cell retention. *LIPSS* and *nano-pillars* instead present only moderate water repellency but features that are similar in size to *E*. *coli* and *S*. *aureus*. Hydrophilic *LIPSS* present the same morphology as hydrophobic *LIPSS* but do not display water repellency at all, allowing direct assessment of the influence of wettability on bacterial retention.

### Bacterial retention

Dominance of surface morphology over wettability is evident for *E*. *coli*, with water repellency playing a very limited role in influencing initial cell retention. This is seen in both the large residual bacteria count for *spikes* and the relatively minor difference in residual bacteria count between hydrophobic and hydrophilic *LIPSS* (Fig. 4). It is instead clear that surface morphology strongly influences *E*. *coli* retention. *LIPSS* and *nano-pillars*, both with a density of peaks above the critical value of 1 µm^−2^, display marked antibacterial performance, even after a second contamination cycle for *LIPSS*. *Spikes*, with a density of peaks well below the critical value, instead perform poorly. Mirror-polished surfaces also perform poorly for *E*. *coli*, representing a worse outcome than current industrial practice. These results highlight the importance of surface structure over average surface roughness and hydrophobicity for *E*. *coli*. This is consistent with the findings of Epperlein *et al*.^17^, who observed a large reduction in cell retention on *LIPSS*-covered stainless steel surfaces compared to polished specimens.

The response of *S*. *aureus* appears to be more strongly influenced by wettability and average surface roughness. In this case, both mirror-polished and laser-induced *spikes* provide improvements over control samples (Fig. 4), the former achieving relatively large reductions in cell retention. In contrast to *E*. *coli*, the superhydrophobicity of *spikes* may have influenced *S*. *aureus* attachment and self-cleaning of the surface. The low surface roughness of mirror-polished samples (*S*_*a*_ of 30 nm) also leads to improvements. Based on these observations, *LIPSS* and *nano-pillars* perform well due to both low surface roughness (*S*_*a*_ of 90 nm and 60 nm, respectively (Table 3)) and moderate hydrophobicity. Epperlein *et al*.^17^ reported preferential attachment of *S*. *aureus* on *LIPSS* with a static water contact angle of 79°. This apparent difference may have been due to higher wettability and static bacteria cultivation. Fadeeva *et al*.^18^ observed increases in *S*. *aureus* retention on superhydrophobic laser-treated titanium surfaces with large 10–20 µm features compared to polished samples. These results are consistent with the present study, where laser-induced *spikes* performed worse than mirror-polished samples. Cunha *et al*.^34^ instead observed reductions in *S*. *aureus* biofilm formation on *LIPSS* and *nano-pillars* compared to polished titanium samples. Similar results were obtained in the present study for *LIPSS* on stainless steel.

## Conclusion

Though the capacity to create passive antibacterial surfaces via ultrashort pulsed laser irradiation has now been demonstrated on several occasions, the underlying mechanisms leading to this behavior have not always been distinct. The introduction of a new approach for evaluating antibacterial performance based on existing ISO standards represents an important step towards a unified approach for comparing and verifying outcomes achieved with new surface treatments and structure types. In the present study, *E*. *coli* and *S*. *aureus*, chosen to represent gram-negative and gram-positive bacteria strains with dissimilar geometry, are shown to exhibit very different responses to laser-treated surfaces after two hours of immersion in bacterial broth. Retention of the former depends almost exclusively on the relationship between surface morphology and cell dimensions, while that of the latter exhibits dependence on wettability and average surface roughness. Neither very low surface roughness nor superhydrophobicity alone are therefore appropriate for reducing bacterial retention over a wide spectrum of cell types. *LIPSS* and *nano-pillars* obtained via ultrashort pulsed laser irradiation have been shown to provide characteristics that are appropriate for both *E*. *coli* and *S*. *aureus*. Both *LIPSS* and *nano-pillars*, comprising fine features similar in size to bacterial cells, limit available attachment points while exhibiting low surface roughness (*S*_*a*_: 60–90 nm) and moderate hydrophobicity (static water contact angle: 119–140°). It is possible that *LIPSS* produced with shorter wavelengths, having yet finer features, could potentially improve performance with regards to *S*. *aureus*. Further investigation is therefore required into laser-treatment strategies that produce surfaces with smaller features and a density of peaks above 4 µm^−2^. Near infrared ultrashort pulsed laser irradiation, however, currently remains the most feasible solution and provides improvements over present-day practices. There are still a range of challenges to be addressed, including upscaling to match present-day manufacturing throughput, verification of longevity and wear resistance in real industrial environments, and longer bacterial retention observation times to more accurately reflect long-term biofouling. With appropriate development, however, ultrashort pulsed laser technology is likely to become a strong candidate for high-throughput production of antibacterial surfaces.
